# Personality traits and night eating syndrome in women with bulimia nervosa and binge eating disorder

**DOI:** 10.1007/s40519-021-01221-5

**Published:** 2021-05-31

**Authors:** Natasha D. Melunsky, Francesca Solmi, Zoë Haime, Sarah Rowe, Virginia V. W. McIntosh, Janet D. Carter, Jennifer Jordan

**Affiliations:** 1 Division of Psychiatry, University College London, 6th Floor, Maple House, London, W1T 7NF UK; 2 University of Canterbury, Christchurch, New Zealand; 3 University of Otago, Christchurch, New Zealand; 4 Canterbury District Health Board, Christchurch, New Zealand

**Keywords:** Binge eating disorder, Bulimia nervosa, Eating disorders, Night eating syndrome, Personality, Trait

## Abstract

**Purpose:**

Previous research suggests that eating disorders may be associated with certain personality profiles; however, there is limited research investigating associations with night eating syndrome (NES). This research suggests harm avoidance personality trait is higher in NES individuals than in the general population, however, evidence of associations with other personality traits is inconsistent. To understand which personality traits are associated with NES symptoms, the current study aimed to improve understanding of the relationship between NES symptoms and a range of personality traits, addressing limitations in the earlier literature in this area by controlling for common confounders.

**Methods:**

Baseline data were analysed from an outpatient psychotherapy trial for 111 women with bulimia nervosa or binge eating disorder. Pre-treatment measures of personality traits (measured with the Temperament and character inventory—revised) and NES symptoms (measured with the Night eating questionnaire) were used. Regression analyses tested associations between these variables, adjusting for potential confounders, including age and ethnicity.

**Results:**

Low cooperativeness scores were associated with greater NES symptoms in the multivariable model (mean difference: − 0.10, 95% confidence intervals: − 0.20 to − 0.01, *p* = 0.033). There was weak evidence of associations between both high harm avoidance and low self-directedness personality traits and greater NES symptoms.

**Conclusions:**

This study adds to the limited research measuring associations between a range of personality traits and NES, addressing limitations of previous research. Weak evidence for an association between high harm avoidance and low self-directedness and increased NES symptoms was found. A novel association was found between low cooperativeness and greater NES symptoms. Further research is needed to validate its presence in those with and without comorbid eating disorders and to examine the relative change in NES, eating disorder symptoms and personality scores in treatments focusing on cooperativeness.

**Level of evidence:**

Level IV (cross-sectional data from a randomised controlled trial, CTB/04/08/139).

**Supplementary Information:**

The online version contains supplementary material available at 10.1007/s40519-021-01221-5.

## Introduction

Night eating syndrome (NES) was first described by Stunkard in the 1950s [1], but has only gained more prominence since its introduction in the Diagnostic and Statistical Manual of Mental Disorders, Fifth Edition (DSM-5; American Psychiatric Association) [2] under the other specified feeding or eating disorder (OSFED) umbrella category. It is characterised by recurrent episodes of night eating defined as eating more than 25% of one’s daily caloric intake after awakening from sleep or after the evening meal, not better explained by external influences, social norms, another psychiatric disorder, medical disorder or effect of medication. Typically, individuals with NES believe they must eat to sleep and are aware of consuming food at night, unlike the parasomnia sleep-related eating disorder. The night eating must also cause significant distress and/or impairment in functioning [3, 4]. Research typically assesses NES using a proposed diagnostic criteria based on these symptoms, or measures NES symptoms using a continuous measure, scores above the cut-off indicating the likely presence of NES. Since NES was only included in the DSM-5, research on its aetiology is limited.

Although NES is defined as a separate diagnosis within the OSFED classification it is often comorbid with other eating disorder (ED) diagnoses, particularly bulimia nervosa (BN) and binge eating disorder (BED). Up to 52% of people with BED and 35% of those with bulimia nervosa meet criteria for NES, compared with around 10% of people with anorexia nervosa (AN) [5] and 1.5% of the general population [3]. Previous research suggests that the presence of NES comorbid with other eating disorders, such as BED, increases impairment, such as anxiety [6].

Individuals who have more symptoms of NES have been reported to have greater psychological morbidity, including higher stress [7], higher impulsivity [8, 9], lower self-esteem, greater functional impairment [10], and more comorbid psychiatric mood and anxiety disorders [11, 12]. Emotional eating, defined as eating in the absence of hunger or in response to negative emotional cues such as anxiety [13, 14], is also common in people with NES [15].

Previous research suggests that EDs might be associated with certain personality profiles [16]. One way in which this has been investigated is using the Temperament and character inventory (TCI) based on Cloninger’s Psychobiological model of Personality; a dimensional measure of seven personality traits related to temperament and character [17]. Research suggests that certain TCI personality traits, such as harm avoidance, could increase a person’s risk of developing an ED [16], and possibly predict prognosis and drop-out in ED interventions [18]. Furthermore, cognitive-behavioural therapy (CBT) for BN was found to decrease harm avoidance and increase self-directedness [19]. The relationship between CBT and personality traits is bi-directional. Improvement in these personality traits may predict successful outcome in CBT, and in turn, CBT may affect positive change in elements that contribute to these traits [20]. The presence of particular ED symptoms, such as purging behaviour, may moderate the correlation between personality traits and EDs [21, 22]. Understanding which personality traits are associated with NES may pave the way to more targeted aetiological research, necessary to identify potential targets for intervention.

To date, only two studies have investigated personality traits in individuals with NES, finding that those with NES report higher harm avoidance [9, 23] and lower self-directedness [23]. The two existing studies explore the relationship between personality traits and NES using different measures (a diagnostic criteria that has been proposed in previous research [9]; Night eating questionnaire (NEQ) as a continuous measure of NES symptoms [23]). These studies have a number of limitations. Both had very small NES sample sizes (*n* = 18; [23], and *n* = 24; [9]), which may have resulted in type I and II errors. One of these studies [23] included only obese individuals with a very high body mass index (BMI), which reduces the generalisability of findings. The other study [9] included only individuals who engaged in nocturnal eating after falling asleep, which is not a specific requirement of NES, and may have also included those with a parasomnia, sleep-related eating disorder (SRED). Previous studies have not appropriately accounted for confounding factors, adjusting for only BMI and age. This may have led to biased estimates, since factors such as ethnicity and employment are associated with eating behaviours [24, 25] and personality traits [26]. To address these limitations and further investigate the associations between personality traits and NES, the current study investigated the cross-sectional association between a range of personality traits and NES symptoms in a sample of treatment-seeking women with binge eating disorder or bulimia nervosa, adjusting for multiple confounders, whilst using a dimensional measure of night eating.

## Method

### Sample

This study is a secondary analysis of pre-treatment data from a previously conducted randomised clinical trial, the Binge Eating Psychotherapy (BEP) study [19]. The BEP trial was conducted in the Clinical Research Unit at the University of Otago (Christchurch, New Zealand) and compared three psychotherapies for BN and BED: standard cognitive-behavioural therapy (CBT), schema therapy, and nutrition and appetite-focused CBT. Recruitment into the study started in 2005 and ended in 2010. Participants were assessed at six time points: at study entry (pre-treatment), at the end of the intervention, and at four yearly follow-ups after treatment ended. One hundred and twelve participants were recruited into the study by advertisements or referral by general practitioners or other health professionals. Participants were women, 16 years or older, with regular binge eating (meeting current DSM-IV criteria for BN or BED), and a body mass index (BMI) greater than 17.5. Participants were excluded if they had current severe psychiatric conditions (major depression, psychoactive substance dependence, bipolar I disorder or schizophrenia) or suicidality requiring immediate treatment, if they had received CBT or schema therapy to treat binge eating problems in the past year or were currently taking psychoactive medication.

Full details of the trial have been previously reported [19]. The clinical trial was approved by the Upper South A Regional Ethics Committees, New Zealand (CTB/04/08/139). The current study used pre-treatment cross-sectional data, including participants with complete data on all variables of interest.

### Measures

#### Structured clinical interview for DSM-IV

Socio-demographic characteristics including age, ethnicity (New Zealand European, Māori, Non-New Zealand European, Asian, Other) and employment (on benefits other than employment benefit, unemployed, student, housewife, employed), which were collected in the clinician assessment immediately prior to conducting the Structured Clinical Interview for DSM-IV (SCID; [27]).

Psychiatric comorbidity, specifically the presence of any anxiety disorder or major depressive disorder in the past month, was assessed using the SCID [27].

#### Night eating questionnaire

Night eating syndrome symptoms were assessed using the self-report Night eating questionnaire (NEQ; [28]). The NEQ has 14 items measuring six core factors: level of morning hunger, time of first meal, percentage of caloric intake after evening meal, initial insomnia, frequency of awakenings after going of sleep, and frequency of nocturnal eating episodes. Questions are scored using a Likert-type scale (ranges vary across questions) and are summed to derive a total score ranging from 0 to 52, with higher scores indicating greater NES severity (supplementary material A).

The NEQ is a dimensional assessment of NES severity, and is often used as a screening tool for NES due to its adequate reliability (alpha = 0.70), and research supporting its validity, such as its factor structure, in diverse samples [29, 30]. Allison suggested using a higher threshold of 30 to avoid false positives, however, most other studies have used a cut-off of 25, with higher scores indicating the likely present of NES. A cut-off of 25 was used in this study. [29].

#### Temperament and character inventory—revised

Personality traits were measured using the Temperament and character inventory**—**revised (TCI-R; [17]). The TCI-R is a 240 item self-report questionnaire measuring four temperament dimensions (novelty seeking, harm avoidance, reward dependence, persistence) and three character dimensions (self-directedness, cooperativeness, self-transcendence). For each dimension, participants rate how well each item describes the way they usually act or feel using a Likert-type scale from 1 (definitely false) to 5 (definitely true). Individual items are summed to derive a total score for each dimension, where higher scores indicate greater strength of a trait.

The TCI-R was used as it has been shown to be a reliable and valid tool, with Cronbach’s alpha ranging from 0.82 to 0.90 [31, 32], and divides personality up into small trait areas to better understand aetiology and potentially to inform intervention.

#### Eating disorders examination-12

ED diagnosis was controlled for and assessed at pre-treatment using the Eating Disorders Examination-12 (EDE-12; [33]). The EDE is a 12-item structured clinical interview that measures the severity of ED psychopathology through the frequency and regularity of eating, restricting, and purging behaviours. Participants were classified as having BN purging type (BN-P), BN non-purging type (BN-NP), or binge eating disorder (BED, see supplementary material B).

### Data analyses

Analyses were conducted using Stata14 [34]. Sample characteristics were described using frequencies with proportions, and means with standard deviations.

To test the association between personality traits and NES symptoms, univariable and multivariable linear regression models were used. A univariable model and two multivariable models were calculated progressively adjusting for age, ethnicity, employment status, diagnosis of BN-P, BN-NP or BED (multivariable model 1), and psychiatric comorbidity (multivariable model 2). Previous studies have shown that greater psychiatric comorbidity may be associated with NES symptoms [7, 11, 12]. Certain temperament and character traits have been found to be risk factors for depression and anxiety. It is also possible that the latter could exacerbate temperament and character traits [35], so we controlled for psychiatric comorbidity in the current study. As this analysis is cross-sectional and temporality cannot be inferred, depression and anxiety were adjusted for in a separate model.

To test the potential impact of purging status in BN on the association between personality traits and NES symptoms, two multivariable models were calculated progressively adjusting for the confounders: age, ethnicity, employment status, diagnosis of BN-P or BN-NP (multivariable model 3) and psychiatric comorbidity (multivariable model 4).

## Results

### Sample

In total, 112 women were enrolled in the BEP study. One woman had missing NEQ data and, therefore, was excluded from the analysis leaving 111 participants (99%). Table 1 shows the sociodemographic characteristics of participants. The majority of participants were of New Zealand European ethnicity (*n* = 75, 67.0%), 46.4% were married or co-habiting for one or more year (*n* = 52), and 54.5% were employed (*n* = 61). The mean age of participants was 35 years (standard deviation (SD) = 12.6), and mean BMI was 30.0 (SD = 7.8), which ranged from 18 to 50.

**Table 1:** Socio-demographic and clinical characteristics of the study sample

	Total *N* (%)	^a^Personality traits M (SD)						
		Novelty seeking Total	Harm avoidance Total	Reward dependence Total	Persistence Total	Self-directedness Total	Cooperativeness Total	Self-transcendence Total
Ethnicity								
NZ European	74 (66.7)	107 (15.1)	106 (20.6)	102 (12.4)	113 (23.2)	129 (19.9)	135 (15.2)	60 (18.4)
Maori	11 (9.9)	108 (16.4)	103 (19.7)	97 (16.8)	111 (25.9)	132 (17.5)	140 (8.9)	61 (13.5)
Non NZ European	19 (17.1)	103 (19.2)	112 (19.9)	108 (17.1)	103 (21.1)	121 (21.2)	136 (16.6)	65 (12.7)
Asian	4 (3.6)	109 (10.8)	103 (19.0)	104 (8.2)	105 (24.6)	125 (17.0)	132 (5.2)	62 (9.5)
Other	3 (2.7)	95 (11.9)	93 (15.0)	109 (18.0)	122 (17.6)	127 (21.9)	141 (22.2)	55 (10.5)
Age *								
16–25	31 (27.9)	110 (13.9)	110 (18.3)	102 (13.7)	112 (23.6)	125 (14.1)	131 (15.5)	59 (16.6)
26–35	30 (27.0)	111 (15.6)	103 (19.4)	106 (15.6)	113 (22.2)	130 (21.1)	141 (14.5)	60 (14.7)
36–45	26 (23.4)	101 (18.2)	110 (22.6)	98.8 (10.5)	112 (20.6)	121 (19.9)	137 (13.4)	64 (15.8)
46–55	16 (14.4)	101 (14.8)	103 (21.3)	104 (13.1)	109 (22.1)	131 (21.2)	132 (15.1)	59 (15.6)
56–65	8 (7.2)	106 (8.9)	95 (15.3)	107 (18.0)	102 (34.9)	144 (22.1)	142 (11.9)	69 (26.7)
Employment								
On benefit other than unemployment benefit	9 (8.1)	101 (19.5)	114 (16.3)	97 (9.6)	94 (18.8)	128 (11.7)	137 (16.7)	74 (18.0)
Unemployed	3 (2.7)	100 (20.1)	102 (10.3)	108 (16.5)	102 (40.1)	139 (10.6)	141 (11.1)	60 (8.3)
Student	26 (23.4)	111 (12.1)	107 (18.8)	104 (14.8)	110 (23.3)	127 (15.9)	135 (16.3)	59 (17.3)
Homemaker/full time parent	13 (11.7)	99 (14.6)	114 (17.8)	107 (15.9)	110 (25.3)	123 (17.3)	143 (9.3)	59 (12.2)
Employed	60 (54.1)	108 (15.2)	103 (21.6)	103 (13.5)	115 (21.6)	129 (22.9)	135 (14.9)	61 (16.7)
Marital status								
Married or living together 1 + years	52 (46.9)	105 (14.6)	104 (21.2)	104 (15.0)	111 (23.2)	128 (21.4)	136 (13.7)	59.5 (17.1)
Separated	8 (7.2)	104 (20.4)	105 (22.1)	99 (6.1)	109 (26.0)	133 (15.4)	141 (14.9)	67 (17.2)
Divorced	6 (5.4)	94 (12.9)	112 (4.4)	106 (12.4)	94 (21.5)	129 (13.8)	134 (15.3)	56 (8.4)
Widowed	1 (0.9)	133 (.)	110 (.)	116 (.)	99 (.)	115 (.)	140 (.)	87 (.)
Never married	44 (39.6)	110 (15.3)	108 (20.4)	102 (13.9)	114 (22.4)	126 (19.7)	135 (16.3)	62 (16.5)
Eating disorder diagnosis								
BN-P	29 (26.1)	108 (16.7)	104 (18.9)	103 (15.2)	116 (20.7)	126 (13.8)	138 (13.0)	60 (15.0)
BN-NP	29 (26.1)	107 (16.1)	108 (20.2)	107 (10.9)	115 (19.1)	130 (23.7)	138 (15.9)	64 (19.3)
BED	53 (47.8)	105 (15.2)	106 (21.1)	101 (14.3)	106 (25.3)	127 (20.4)	134 (15.1)	60 (16.0)
Major depressive disorder diagnosis								
Yes	30 (27.0)	104 (15.7)	110 (15.4)	100 (14.0)	113 (27.1)	124 (13.5)	137 (14.2)	63 (19.4)
Any anxiety disorder diagnosis (in past month)								
Yes	53 (47.8)	104 (16.7)	114 (18.5)	101 (14.3)	110 (22.8)	123 (18.6)	131 (15.1)	63 (18.2)
BMI*								
16–25	44 (39.6)	106 (17.7)	108 (21.2)	104 (13.4)	113 (19.8)	128 (20.4)	138 (16.1)	61 (15.2)
26–35	42 (37.8)	107 (14.9)	103 (17.0)	103 (13.6)	113 (20.7)	126 (18.4)	135 (14.4)	61 (18.4)
36–45	23 (20.7)	107 (14.5)	107 (23.2)	102 (15.5)	105 (31.0)	131 (21.7)	136 (13.8)	62 (17.1)
46–55	1 (0.9)	113 (.)	96 (.)	124 (.)	82 (.)	129 (.)	130 (.)	53 (.)
56–65	1 (0.9)	100 (.)	140 (.)	95 (.)	89 (.)	103 (.)	129 (.)	59 (.)
^b^Night eating syndrome severity	Mean (SD)							
BN-P	17.6 (6.8)							
BN-NP	19.8 (8.6)							
BED	16.5 (7.2)							

*BED* binge eating disorder, *BN* bulimia nervosa, *BNNP* bulimia nervosa non-purging, *BNP* bulimia nervosa purging, *NZ* New Zealand, *SD* standard deviation

^a^Personality traits as measured by totals of sub-scales in the Temperament and character inventory—revised (TCI-R)

^b^Night eating severity as measured by the Night eating questionnaire total score

Comorbid psychiatric disorders were common in the sample; 30 women (27%) had a diagnosis of major depressive disorder (MDD) and 53 (47.8%) had an anxiety disorder in the past month. Around half (*n* = 58, 52.2%) had a diagnosis of BN (26.1% BN-P, and 26.1% BN-NP), and the mean duration of the ED was 12.1 years (SD = 10.4) for BN and 17.6 years (SD = 14.7) for BED.

Personality traits and night eating scores are shown in Table 1 and in supplementary material C. The mean length of time participants reported NES symptoms was 60.1 days (SD = 96.3). NEQ Total scores were similar across diagnoses. The mean NEQ Total score was 17.3 (SD = 7.5), with 25.2% meeting criteria for NES, using the NEQ-screening cut-off of 25 [26].

### Association between personality traits and night eating

As shown in Table 2, there was no evidence of an association between novelty seeking, reward dependence, persistence, or self-transcendence scores and NEQ scores, in univariable or multivariable models.

**Table 2:** Standardised association between TCI-R personality traits and night eating syndrome symptoms

*N* = 111	Unadjusted coefficient (95% CI; *p* value)	^a^Adjusted coefficient (95% CI; *p* value)	^b^Adjusted coefficient (95% CI; *p* value)
Novelty seeking	− 0.71 (− 2.15 to 0.72; *p* = 0.325)	− 0.66 (− 2.12 to 0.81; *p* = 0.378)	− 0.42 (− 1.88 to 1.04; *p* = 0.570)
Harm avoidance	0.62 (− 0.81 to 2.06; *p* = 0.392)	0.99 (− 0.44 to 2.43; *p* = 0.173)	0.32 (− 1.24 to 1.88; *p* = 0.685)
Reward dependence	0.56 (− 0.88 to 1.99; *p* = 0.443)	0.36 (− 01.08 to 1.80; *p* = 0.625)	0.76 (− 0.69 to 2.21; *p* = 0.300)
Persistence	0.72 (− 71 to 2.16; *p* = 0.318)	0.43 (−1.07 to 1.94; *p* = 0.569)	0.45 (− 1.03 to 1.93; *p* = 0.548)
Self-directedness	− 1.04 (− 2.46 to 0.39; *p* = 0.151)	− 1.18 (− 2.61 to 0.25; *p* = 0.104)	− 0.76 (− 2.24 to 0.71; *p* = 0.308)
Cooperativeness	− 1.36 (− 2.77 to 0.06; *p* = 0.060)	− 1.54 (− 2.95 to − 0.12; *p* = 0.033)	− 1.08 (− 2.58 to 0.42; *p* = 0.156)
Self-transcendence	0.70 (− 0.73 to 2.14; *p* = 0.333)	0.76 (− 0.69 to 2.20; *p* = 0.301)	0.61 (− 0.82 to 2.03; *p* = 0.403)

^a^Adjusted for the confounders: age, ethnicity, employment status, diagnosis of bulimia nervosa or binge eating disorder

^b^Adjusted for the confounders: age, ethnicity, employment status, diagnosis of bulimia nervosa or binge eating disorder, BMI, any anxiety disorder in the past month, major depressive disorder in past month

When controlling for age, ethnicity, employment status and diagnosis the association between greater harm avoidance and more NES symptoms was weak (standardised mean difference (MD) = 0.99, 95% confidence intervals (CI): − 0.44 to 2.43, *p* = 0.173). This association, however, weakened further and was not considered significant when also controlling for psychiatric comorbidity in multivariable model 2 (standardised MD = 0.32, 95% CI − 1.24 to 1.88; *p* = 0.685). As shown in Table 3, when controlling for purging status within BN diagnosis in multivariable model 4, strength and significance of the association increased slightly (standardised MD = − 2.00, 95% CI − 4.73 to 0.73; *p* = 0.146).

**Table 3:** Standardised association between TCI-R personality traits and night eating syndrome symptoms in BN

*N* = 111	^a^Adjusted coefficient (95% CI; *p* value)	^b^Adjusted coefficient (95% CI; *p* value)
Novelty seeking	− 0.14 (− 2.16 to 1.87; *p* = 0.887)	0.14 (− 1.95 to 2.23; *p* = 0.892)
Harm avoidance	− 0.01 (− 2.31 to 2.29; *p* = 0.991)	− 2.00 (− 4.73 to 0.73, *p* = 0.146)
Reward dependence	− 0.08 (− 2.34 to 2.18; *p* = 0.945)	0.23 (− 2.07 to 2.53, *p* = 0.842)
Persistence	0.59 (− 1.88 to 3.05; *p* = 0.635)	1.06 (− 1.50 to 3.62, *p* = 0.409)
Self-directedness	− 0.63 (− 2.73 to 1.47; *p* = 0.550)	0.24 (− 2.03 to 2.51, *p* = 0.835)
Cooperativeness	− 1.85 (− 3.97 to 0.27; *p* = 0.085)	− 1.4 (− 3.70 to 0.81, *p* = 0.203)
Self-transcendence	1.49 (− 0.54 to 3.53; *p* = 0.147)	1.28 (− 0.74 to 3.31, *p* = 0.209)

^a^Adjusted for the confounders: age, ethnicity, employment status, and purging behaviour

^b^Adjusted for the confounders: age, ethnicity, employment status, purging behaviour, any anxiety disorder in past month, and major depressive disorder in past month

As seen in Table 2 in the univariable model, a weak association was observed between higher self-directedness scores and fewer NES symptoms (standardised MD = − 1.04, 95% CI − 2.46 to 0.39, *p* = 0.151). This association was slightly stronger in multivariable model 1, when accounting for sociodemographic confounders of age, ethnicity, employment status and diagnosis (standardised MD = − 1.18, 95% CI − 2.61 to 0.25, *p* = 0.104). In multivariable model 2, when controlling for psychiatric comorbidity the size and strength of the association weakened (standardised MD: − 0.76, 95% CI − 2.24 to 0.71, *p* = 0.156). In Table 3, no association was found between self-directedness and NES symptoms when controlling for purging behaviour in multivariable model 4 (standardised MD: 0.24, 95% CI − 2.03 to 2.51, *p* = 0.835).

As shown in Table 2, after standardising, a one-standard deviation increase in the cooperative dimension of the TCI-R was associated with a 1.36 reduction in mean NEQ total score, illustrating a weak to moderate association (95% CI − 2.77 to 0.06, *p* = 0.060). After adjustment for confounders in multivariable model 1, there was greater evidence of an association between higher cooperativeness scores and fewer NES symptoms, albeit the size of the latter remained unchanged: (standardised MD = − 1.54, 95% CI − 2.95 to − 0.12, *p* = 0.033). The strength of the association weakened when also controlling for psychiatric comorbidity in multivariable model 2 (standardised MD = − 1.08, 95% CI − 2.58 to 0.42, *p* = 0.156, see Table 2), and controlling for purging behaviour in multivariable model 4 (standardised MD: 1.44, 95% CI − 0.3.70 to 0.81, *p* = 0.203, see Table 3).

## Discussion

This study investigated the cross-sectional association between personality traits and NES symptoms in treatment-seeking women with BN or BED. Previous studies found an association between harm avoidance, and self-directedness, and NES symptoms [9, 23], which was weakly supported by the current study. In contrast, the current study found participants with lower cooperativeness scores reported more NES symptoms, which is a novel finding. Although previous studies have reported correlations between low cooperativeness and higher ED symptoms in AN, BN and BED, this association was suggested to be stronger in purging than non-purging subtypes [21, 22]. However, when controlling for purging behaviour in multivariable model four, the strength of the association did not change, indicating that the association was not present in both BN purging and non-purging subtypes in this sample.

Several hypotheses may explain the novel association between low cooperativeness and higher NES symptom scores. Cooperativeness is a character trait describing the degree to which a person is generally agreeable in their relationships with other people, indicated by their broadness of social concern and flexibility of judgement [31, 36]. Those with a low score on the TCI-R cooperativeness dimension are described as intolerant, critical, having low empathy and primarily looking out for themselves [17, 22]. Low cooperativeness has been reported to be common to all ED types [36, 37]. It is, therefore, possible that low cooperativeness may represent a transdiagnostic risk factor for all ED diagnoses, including NES.

In particular, low compassion (a sub-domain of cooperativeness) is observed across all EDs and is associated with poorer therapeutic outcomes [38, 39]. Previous research has found that interpersonal psychotherapy, targeting elements of cooperativeness, has demonstrated efficacy in reducing binge eating episodes and inducing weight stabilisation in those with BED [40, 41]. Taken together these findings suggest that compassion may be a potential specific target for NES treatment.

Findings from the current study may also be explained by the presence of psychiatric comorbidities. The strength of the association between cooperativeness and NES symptoms is reduced when adjusting for anxiety or depression diagnoses. This association has also been shown in a study by Duarte et al. [42] who found that a psychoeducation intervention targeting the cooperativeness personality trait, reduced binge eating whilst improving depression and stress scores. This suggests that psychiatric comorbidities may be on the associative pathway between cooperativeness and binge eating disorders.

The current study found a weak association between low self-directedness and NES symptoms. This association is consistent with the findings of one study examining the association between personality and NES which, after controlling for BMI and age, found NES symptoms increased as self-directedness scores reduced [23]. Previous evidence has also shown a correlation between low self-directedness and increased AN, BN, BED, and symptoms of other psychiatric diagnoses [37, 43]. Additionally, a CBT trial with individuals with BN found evidence that higher self-directedness resulted in better symptom outcomes [20]. Furthermore, CBT has been shown to actively improve self-directedness scores and ED behaviours [20, 49].

In contrast to previous studies that have reported strong associations between harm avoidance and NES [36, 44], in the current study there was only weak evidence for an association between harm avoidance and NES symptoms. Furthermore, research into EDs has consistently found high harm avoidance in individuals diagnosed with an ED and also most other psychiatric diagnoses [22, 43]. Previous research suggests harm avoidance may predict prognosis and drop-out in ED interventions [18].

The primary explanation to describe weak associations between NES symptoms and low self-directedness and high harm avoidance in the current study is that these traits are common in most psychiatric disorders including EDs. The sample in the current study had elevated rates of psychiatric comorbidities as well as their ED and, therefore, small differences in trait scores between those with and without NES are likely to be difficult to detect within the already high levels evident within this sample. Self-directedness and harm avoidance, albeit less so, had apparent clinical relevance when compared to a general population sample [45], strengthening this explanation.

Therefore, it is possible self-directedness and harm avoidance could be targeted in NES interventions in the future, however, due to limited evidence of the association between these traits and NES thus far, further research on the aetiology of NES is needed. Future studies should be conducted in those with NES alone compared to those with NES comorbid with EDs from the general population to allow for more variance in the sample, and to account for the higher strength of personality traits typical of the ED population.

Strengths of this study include that this is the largest study to date investigating the association between personality traits and NES symptoms. Previous studies used a high BMI and control population, and a NES and control population respectively [9, 23]. The current study explored NES for individuals with either BN or BED diagnoses. Examining NES in these participants with EDs adds to the evidence base of the presence of nocturnal eating in different populations. Greater understanding of the associations of personality and NES in those with EDs has potential to inform development of interventions to reduce NES within a clinically diagnosed population.

The NEQ was used, which has been validated across multiple populations [29, 30] and provides a continuous score for NES symptoms which enabled the full spectrum of symptomatology to be captured, reducing the likelihood of spurious associations that might emerge from dichotomising variables [46, 47]. The current study adjusted for multiple confounders that have been previously found to be associated with both personality and NES, including psychiatric diagnoses from structured clinical interviews.

Despite these strengths, study results should be interpreted in light of some limitations. The paucity of previous similar studies meant there was insufficient information for a priori power calculations. Wide confidence intervals for some variables indicate heterogeneity within the sample. It is possible then that the sample size in this study may have been underpowered to detect effects that might be present, as such raising the chance of Type II error. Furthermore, that all in the sample had diagnoses of BN or BED limits the generalisability of the findings. For example, previous studies have reported that individuals with comorbid NES and BED had higher trait anxiety, than those with NES without comorbidity [6].

Personality is believed to be relatively stable during a person’s lifetime [48], so it could be hypothesised that NES may be a consequence of particular personality traits. There is also evidence that personality traits and symptoms can improve after successful ED treatment [49]. Additionally, due to the current cross-sectional study design, it was not possible to ascertain the temporal direction of associations, which is necessary to begin to establish causality. Although the NEQ has the advantage of dimensional data for analyses, the study did not use clinical interviews to establish NES diagnoses, which could be perceived as a shortcoming [50]. Longitudinal designs using structured interviews to assess NES are now needed to explore whether personality traits might be aetiologically relevant for NES.

## Conclusions

In conclusion, this paper aimed to improve understanding of the relationship between NES symptoms and personality traits, addressing limitations in earlier literature in this area by controlling for common confounders. There was a novel finding of an association between low cooperativeness and higher NES symptoms, and support (albeit weak) for previously identified associations between high harm avoidance and low self-directedness and increased NES symptoms for women with BN and BED. The study did not find any personality trait differences between purging and non-purging BN. Greater knowledge of the influence of personality traits in NES in those with EDs is important as it may point to potential treatment targets to improve treatment outcomes for this group. For example, psychotherapies such as IPT and compassion-based therapies that focus on improving emotional understanding of self and others and improving interpersonal functioning, and in doing so, addressing cooperativeness, may be particularly useful with this group.

Further longitudinal research is needed to examine the trajectory of change in NES symptoms alongside change in personality traits in treatments that do and do not address those traits in people with NES and other EDs.

## What is already known on this subject?

Only two previous studies have investigated the association of personality traits with NES*.* These studies reported higher harm avoidance and lower self-directedness in people with NES but the findings were limited by small sample sizes and reduced generalisability. Understanding which personality traits are associated with NES may pave the way to more targeted aetiological research.

## What does the study add?

This study provides evidence for a novel association between cooperativeness personality trait and NES. It has implications for further research and potential targets for treatment.

## Supplementary Information

Below is the link to the electronic supplementary material.

Supplementary file1 (DOCX 29 KB)

## Data Availability

The data that support the findings of this study are available from the corresponding author upon reasonable request.
