# Supplementary material for: Personality traits and night eating syndrome in women with bulimia nervosa and binge eating disorder
Source: Eat Weight Disord. 2021 May 31;27(2):803–12. doi: 10.1007/s40519-021-01221-5 (PMC8933296; doi:10.1007/s40519-021-01221-5)
Supplement: Supplementary file 1 — Supplementary file1 (DOCX 29 KB) [file 40519_2021_1221_MOESM1_ESM.docx]

Supplementary material A

NEQ

Scoring directions: Questions 1-9, which focus on variables occurring before sleep onset, are answered by all participants. Questions 10-12 are answered by those scoring N1-4 on question 9, and 13 and 14 are answered by those scoring N1-4 on question 12. Question 15 is answered by all, and is not added to the total score, but used as a descriptor of the course of the symptoms. Items 1-14 are scored on a 0-4 Likert scale, with the exception of question 7 which has the additional option “check here if your mood does not change during the day”, which is scored as a zero. Items 1, 4 and 14 are reverse-scored, and items 1-12 and 14 are summed. Item 13 is not included in the total score, but is used to rule out the parasomnia, Nocturnal Sleep Related Eating Disorder. Individuals are considered to have NES if their score is 25 or over.

Supplementary material B

The researchers in the BEP study, due to overlap between BN and BED, split participants into three diagnoses. Participants with 24 or more episodes of purging behaviour in three months were considered ‘Bulimia Nervosa Purging’ (BNP), participants with 24 or more episodes of non-purging compensatory behaviour in three months were considered ‘Bulimia Nervosa Non-Purging’ (BNNP), and participants not in those categories, which included no compensatory behaviour as well as subthreshold cases, <24 episodes of purging, were in the binge-eating disorder (BED) category. Diagnosis based on the three BEP categories was used since research suggests there are differences in NES frequency between BN and BED (Olejniczak et al, 2018; Tu, Tseng, & Chang, 2018). Further information describing how the diagnosis categories were created can be found in Jordan et al., (2014).

Supplementary material C

*Personality trait characteristics of the study sample*

| (*N*=111) | **Mean (SD)** |
| --- | --- |
| Novelty Seeking | 106.7 (15.8) |
| Harm Avoidance | 106.0 (20.2) |
| Reward Dependence | 103.7 (13.9) |
| Persistence | 112.3 (23.0) |
| Self-Directedness | 127.7 (19.8) |
| Cooperativeness | 136.7 (14.8) |
| Self-Transcendence | 61.3 (16.6) |
